# Perspectives of Indigenous University Students in Canada on Mindfulness-Based Interventions and their Adaptation to Reduce Depression and Anxiety Symptoms

**DOI:** 10.1007/s12671-023-02087-7

**Published:** 2023-02-21

**Authors:** Shadi Beshai, Sharon M. Desjarlais, Brenda Green

**Affiliations:** 1 Department of Psychology, University of Regina, 3737 Wascana Parkway, Regina, SK S4S0A2 Canada; 2 Indigenous Health, First Nations University, 1 First Nations Way, Regina, SK S4S 7K2 Canada

**Keywords:** Indigenous, Anxiety, Depression, Mindfulness, University students, Cultural adaptation, MBIs

## Abstract

**Objectives:**

Indigenous university students experience high rates of anxiety and depression due primarily to the pernicious and persistent effects of colonialism, racism, and discrimination. Mindfulness-based interventions (MBIs) hold promise, but likely require adaptation to make them culturally relevant for Indigenous peoples. We sought to gather Indigenous students’ perspectives on the consistency and adaptability of MBIs for Indigenous students experiencing symptoms of depression and anxiety.

**Method:**

This three-part longitudinal investigation employed a qualitative design mixed with Indigenous research methods to elicit feedback from students (*n* = 14; *M*_age_ = 28.92) on the acceptability of MBIs and ways to tailor MBIs to make them more consistent with Indigenous cultures and student lifestyles. We subsequently used this feedback to develop an outline for an adapted MBI that was then re-evaluated by the same participants for its cultural relevance and safety.

**Results:**

Indigenous students emphasized the need for the adapted MBI to incorporate (a) traditional Indigenous practices; (b) Indigenous facilitators; (c) holistic conceptualizations of mental health that include spirituality; and (d) practices and methods that could improve flexibility and accessibility of the adapted intervention. Based on this feedback, we presented students with an outline of an adapted MBI tentatively titled *Miyowâyâwin Mindful Wellbeing Program*, which received favorable evaluations by students for cultural consistency and safety.

**Conclusions:**

We confirmed the perceived acceptability and consistency of mindfulness and mindfulness programs with Indigenous cultures. The need for a flexible MBI that centers Indigenous elements and Indigenous facilitators was highlighted by Indigenous participants. This study paves the way for latter steps of the development and subsequent evaluation of the *Miyowâyâwin Mindful Wellbeing Program*.

**Preregistration:**

This study is not preregistered.

Depression and anxiety are experienced by people of all communities, but are particularly prevalent among Indigenous people due to a history of oppression, lingering systemic racism, and intergenerational trauma caused by colonization (Elias et al., 2012). Indigenous students in particular face compounding risk factors, as they face diverse societal challenges while dealing with the stressors and demands of university studies. This compounded stress has a negative impact on their motivation and feelings of self-worth leading to lower academic success (Bailey, 2016; Currie et al., 2012); and thus, it is associated with higher drop-out rates among Indigenous students (Perry, 2009). On-campus mental health services for Indigenous students may seem logical; however, cultural insensitivity, racism, discrimination, and overt abuse in hospitals and clinics have resulted in a lack of trust between patients and health care practitioners, including mental health care (Tang & Browne, 2008). Furthermore, counselling is based on the Western biomedical model which focuses on the absence of disease or illness as constituting a state of health (Gone, 2004). This model runs counter to the Indigenous perspective, which approaches health within a holistic framework with the physical, mental, emotional, and spiritual states in balance (Gone, 2004). Accordingly, culturally appropriate mental health care, which at its core incorporates Indigenous perspectives and knowledge, is needed to increase uptake and engagement among Indigenous people experiencing heightened distress.

According to the Diagnostic and Statistical Manual – 5 (DSM-5), mood disorders can manifest as one of a number of conditions, all of them distinguished by low mood, lack of pleasure, or irritability, accompanied by somatic and cognitive changes (American Psychiatric Association, 2013). Anxiety can manifest as one of several disorders distinguished by the hallmark features of excessive fear in response to a perceived imminent threat and excessive anxiety in anticipation of a future threat (American Psychiatric Association, 2013). The prevalence of depression and anxiety experienced by Indigenous students on Canadian university campuses has been a concern for many years, and the problem appears to be worsening (Glauser, 2017). Not surprisingly, Beiter et al. (2015) found the top areas of concern that were associated with heightened anxiety were related to the academic life of students: academic achievement, succeeding, finding a job, and financial problems. However, the COVID-19 pandemic and the accompanying social isolation and worries about illness have added an extra layer of distress among those already suffering mental health problems (Treleaven, 2020).

Indigenous students appear to have high rates of depression and anxiety compared with members of the general population. A recent study with Indigenous students at the University of Regina (Chahar Mahali et al., 2020) found that 38% and 41% of Indigenous students experienced moderate to severe symptoms of depression and anxiety, respectively. Indigenous students have the added burden of stressors that derive from racial discrimination and the effects of colonialism (Kirmayer et al., 2000; Waldram, 2004). Currie et al. (2012) found that Indigenous students in Canada experienced racism more frequently and in a wider range of situations than African American and Latino Americans in the USA. Furthermore, microaggressions and other subtle forms of everyday racism further exacerbate mental health concerns among Indigenous students (Bailey, 2016).

Mindfulness is defined as bringing awareness to the present moment with a non-judgmental and open stance (Kabat-Zinn, 1994). Theoretically, mindfulness functions to change one’s relationship with present-moment experiences. Mindfulness cultivates the ability to respond skillfully to the experience of distress and to life’s stressors, rather than fall into learned patterns of avoidance, withdrawal, stress, and anxiety (Feldman & Kuyken, 2019). It trains the mind to shift from rash, impulsive reactions when faced with adverse events and feelings to more thoughtful, reasoned responses (Feldman & Kuyken, 2019). Mindfulness-based interventions (MBIs), which have been shown to increase trainees’ capacity to be mindful, can improve metrics of health and well-being, and lower stress and symptoms of anxiety and depression (Regehr et al., 2013). Mindfulness is universally accessible, as attention regulation and cultivation of an accepting attitude are innate capacities shared by all people (Kabat-Zinn, 1994). According to Yellow Bird (2012), mindfulness is an element of traditional Indigenous practice that can reduce the effects of trauma caused by the stresses of colonialism, resulting in greater compassion, emotion regulation, mind–body connection, and the courage to challenge oppression.

As an added benefit, the concept and practice of mindfulness appear particularly consistent with Indigenous cultural worldviews (Le & Gobert, 2015; Le & Proulx, 2015). Components of mindfulness are in harmony with Indigenous cultural practices and ways of being, as they share common themes of balance, bringing awareness to the present moment, interconnectedness, and being virtuous. From the Indigenous perspective, cultural and spiritual values and practices are part of the healing process and are essential in the promotion of mental health (Gone, 2013; Green, 2010; Kirmayer et al., 2003).

Sasakamoose et al. (2016) found that there was a strong similarity between the philosophy of Eastern yoga and mindfulness meditation and the First Nations medicine wheel with its emphasis on holistic health involving elements of spiritual, mental, emotional, and physical well-being. Youth considered components of Eastern practices as important components in the promotion of mental health and well-being in their communities (Sasakamoose et al., 2016). Several researchers advocate for a “culture as treatment” approach where treatment plans incorporate the use of Indigenous cultural practices to promote the health and well-being of the community (Gone, 2013; Green, 2010; LaFromboise et al., 1990; Pomerville et al., 2016; Stewart, 2008; Waldram, 2004). The idea is that the genocide resulting from colonialization has created a cultural disconnect for many Indigenous peoples and that the reaffirmation of cultural identity can strengthen the resiliency of individuals and communities (Kirmayer et al., 2011) and promote healing, restoring balance and harmony to the community (Green, 2010).

Cultural adaptation of evidence-based interventions can be defined as the “systematic modification of an evidence-based intervention protocol to consider language, culture, and context in such a way that it is compatible with the clients’ cultural patterns, meanings, and values” (Bernal et al., 2009, p. 362). There are several arguments that support the cultural adaptation of evidence-based treatments, such as MBIs, among minoritized people. In the first place, since the majority of research on the efficacy of extant mindfulness treatments have been tested among white, majority culture participants, the social validity and acceptability of these treatments among minoritized clients are unclear (Bernal et al., 1995, 2009). Moreover, there is empirical evidence to support the efficacy of adapted intervention protocols over unadapted intervention protocols when used with minoritized patients (Benish et al., 2011).

There is a chasm in the availability of MBIs that are culturally relevant or meaningful for Indigenous university students suffering from the symptoms of depression and anxiety. Given that Indigenous university students experience mental health symptoms at higher rates than the general population (Chahar Mahali et al., 2020; Government of Canada, 2006; Kirmayer et al., 2009; National Collaborating Centre for Aboriginal Health, 2012), the lack of culturally relevant programs (mindfulness or otherwise) is not only emblematic of the gap in mental health services for Indigenous peoples, but it also highlights the issue of social inequity and runs contrary to the ideal of appropriate mental health care for all. This research study is the first known Canadian study in which researchers sought to solicit feedback in the adaptation of a MBI for use with Indigenous peoples.

There is growing interest in the use of MBIs with ethnic-minority populations; however, research into its efficacy for minority groups, and specifically for Indigenous peoples, is slow to accumulate. Furthermore, much of the mindfulness research has been conducted using highly unrepresentative samples of Western, Educated, Industrialized, Rich, and Democratic (WEIRD) people, who are often undergraduate university students, that account for only 12% of the world’s population (Henrich et al., 2010; Proulx et al., 2017). These populations differ on psychological, motivational, and behavioral domains; and therefore, it is inappropriate to generalize research to specific or place-based ethnic minority groups (Henrich et al., 2010). Furthermore, Watson-Singleton et al. (2019) noted the connection between the dearth of MBIs specifically developed to meet the needs of minorities and the underutilization of mental health services by those same minorities. Accordingly, the populations that are most in need of effective and culturally relevant programs are still underrepresented in research more than 20 years after the need for culture-specific programs was identified (Brave Heart, 1998; Sue, 1999).

There is a body of research that suggests MBIs are efficacious for minority groups. For example, the results from three qualitative studies conducted with Black patients on the appropriateness of MBIs, in which interviews were used for gathering data, yielded consistent results (Proulx et al., 2020; Watson-Singleton et al., 2019; Woods-Giscombé & Gaylord, 2014). Woods-Giscombé and Gaylord (2014) interviewed adults who had some experience in mindfulness meditation to identify how the practice could be employed by individuals or community agencies to mitigate the race-based stress experienced by Black people. The researchers concluded that only minor adaptations would be required to enhance the beneficial effects on the health of Black people. In all three studies, participants reported beneficial results from mindfulness practice. The recommendations made by the participants were remarkably similar for all three studies: emphasize benefits to health, relate the mindfulness meditation practices to the spiritual lives of the participants, and employ Black facilitators.

Two studies examined the efficacy of MBIs with Indigenous youth (Le & Gobert, 2015; Le & Proulx, 2015). The challenge that Le and Gobert (2015) undertook was to determine the feasibility of adapting a mindfulness-based suicide prevention program for Native American youth, a group with the highest rate of suicide in the USA. The adaptation was carried out in a two-step approach. First, elders, cultural committees, and school administration were consulted for ways to make the language, spiritual practices, activities, and delivery fit the needs of the target group (Le & Gobert, 2015). Second, the researchers worked with eight youth on a 9-week pilot of the program (Le & Gobert, 2015). Indigenous youth completing the adapted MBI demonstrated less impulsivity, improved self-regulation, and less suicidal ideation (Le & Gobert, 2015). Overall, the youth were receptive and found the program helpful (Le & Gobert, 2015).

Le and Proulx (2015) used the MBI adapted by Le and Gobert (2015) to evaluate the feasibility of an MBI with incarcerated Native Hawaiian youth to improve impulse control and self-regulation. They noted that the higher rates of delinquency, violence, and substance abuse among Native Hawaiian youth were linked to the social disadvantages that result from racial discrimination (Le & Proulx, 2015). The researchers used both self-reporting and biomarkers to determine reduction in stress and improvements related to impulsivity, self-regulation, mind wandering, and mindfulness (Le & Proulx, 2015). They found the program was efficacious in lowering stress, and although the data concerning behavioral changes were not statistically significant, they did show some improvement in self-regulation, attention, and impulse control (Le & Proulx, 2015).

As mentioned, the construct of mindfulness—with the essential components of present-moment, accepting, balanced awareness—transcends cultures and is consistent with Indigenous worldviews and traditions. Unfortunately, several cross-cultural elements of mindfulness were sanitized and erased as a result of Western colonization of the construct and its applications (Van Gordon & Shonin, 2020). For example, traditional and Eastern conceptualizations emphasize the ethical dimension of mindfulness and its potential value as a tool for community-engagement and social justice (Fleming et al., 2022; Nilsson & Kazemi, 2016). By contrast, Western conceptualizations and applications tend to focus more narrowly on personal or individual goals or symptom reduction.

This three-phase study used a mixed-methods design that included Indigenous research, and traditional qualitative and quantitative methods. The goal was to apply a decolonizing lens to the research. The majority of extant literature has been generated *about* Indigenous people by non-Indigenous researchers. The underlying assumption is that it is possible to understand the worldview, the ways of knowing, and the needs of the subjects, and to be able to identify their best interests even after a brief encounter in the context of a research project. Crane et al. (2017) stated that, to ensure the fidelity and integrity of an adapted MBI, there must be a clear understanding of the population’s needs; and failure to address this will undermine the quality of the intervention being delivered. This study intended to draw in, as much as possible, the voices of members of the Indigenous community on the understanding that they would guide the research process, making the end result more culturally relevant.

Specifically, the purpose of this study was to solicit feedback from Indigenous university students in the adaptation of an MBI for anxiety and depression, and to evaluate their perceptions of its cultural relevance and consistency with the concept of mindfulness. This study had four main objectives, in its examination of Indigenous students’ (a) perceptions of the overlap between mindfulness and traditional Indigenous healing methods; (b) perceptions of the consistency of mindfulness and mindfulness interventions with Indigenous practices and cultures; (c) feedback on how existing MBIs could be adapted to make them more in line with Indigenous cultures; and (d) perceptions of acceptability and cultural sensitivity of a hypothetical adapted MBI for Indigenous youth. We hypothesized that participants would find the culturally adapted version of the MBI culturally consistent and acceptable. Cultural consistency is defined here as being aligned with Indigenous practices, including integration of traditional approaches to healing and traditional knowledge into the MBI. Acceptability is defined as the extent to which students find the program sensitive and appropriate to their needs and thus their likelihood of engagement with the program and likelihood of recommending it to others in their community.

## Method

### Participants

We adopted an idiographic approach, and accordingly, opted for a small sample size. The advantage of this approach is that it allows researchers to extract much richer data, treating each participant as an individual while also allowing the opportunity for making comparisons among them (Robinson, 2014). Community leaders from the various communities were consulted in the recruitment process to ensure an ethical and culturally sensitive approach was enacted.

In the current study, we employed a longitudinal design over three phases. In Phase 1, participants completed an online survey designed to gauge personal narratives and understanding of distress (anxiety and depression), and perceptions of the cultural consistency of the concept of mindfulness and MBIs. In Phase 2, and in keeping with Indigenous ways of knowing, we invited participants in Phase 1 to participate in a Talking Circle designed to gather feedback regarding how mindfulness and MBIs can be adapted to be more consistent with Indigenous cultures and student lifestyles. We used feedback gathered in Phases 1 and 2 to create a description of a potential adapted MBI for use among Indigenous students experiencing depression and anxiety. In Phase 3, we solicited perceptions of acceptability and cultural consistency of this adapted program among the same group of participants. Data collection was over a 2-month period from the beginning of February 2021 to April 2021.

Indigenous university students were recruited from the ta-tawâw Student Centre, University of Regina, from First Nations University, and from local Indigenous communities. Participants were recruited by way of in-class announcement, advertising through student organizations, through the ta-tawâw Student Centre and email lists, and through word-of-mouth by community leaders. Participants were compensated with either one credit (only those students enrolled in 100- or 200-level psychology courses) or a $10 gift certificate for their participation in the online surveys and Talking Circle. Informed consent was obtained from all participants prior to their participation in any study-related tasks. The present study was approved by the University of Regina Research Ethics Board (REB #: 2020–154).

Participant inclusion criteria included (a) being 18 years of age or older; (b) self-identifying as First Nations, Inuit, or Métis; (c) registration as a full- or part-time student; and (d) no previous participation in an MBI. Participants who did not meet these criteria were debriefed, compensated as appropriate, thanked, and excluded from analysis.

A total of 14 (*n* = 12 female) Indigenous undergraduate and graduate university student participants were recruited. Ages ranged from 19 to 43 (*M*_age_ = 28.92; *SD* = 6.18). Of the 14 participants, 8 had never participated in psychotherapy, 3 participants had accessed some form of therapy, 2 preferred not to say whether they had engaged in psychotherapy, and 1 participant did not know if they had participated in psychotherapy. All 14 participants who provided responses on the Phase 1 online survey were invited to take part in the Phase 2 Talking Circle via Zoom. Of the 14 participants in Phase 1, 6 participated in the Phase 2 Talking Circle. Ages of the participants in this phase ranged from 19 to 43 (*M*_age_ = 29.67, *SD* = 7.73). A total of 9 (*n* = 8 female) Indigenous undergraduate and graduate university students who partook in Phase 1 also participated in Phase 3. Ages of participants in Phase 3 ranged from 19 to 37 (*M*_age_ = 27.56, *SD* = 5.50). A summary of participant demographics is provided in Table 1.

**Table 1:** Summary of demographics

	Phase 1 (*n* = 14)	Phase 2 (*n* = 6)	Phase 3 (*n* = 9)
Age: *M* (*SD*)	28.92 (6.18)	29.67 (7.73)	27.56 (5.50)
Gender: *n* (%)			
Female	12 (85.7)	4 (66.7)	8 (88.9)
Male	2 (14.3)	2 (33.3)	1 (11.1)
Indigenous status: *n* (%)			
First Nations	12 (85.7)	6 (100)	7 (77.8)
Metis	2 (14.3)		2 (22.2)
Indigenous Nation: *n* (%)			
Cree	10 (71.4)	4 (66.7)	7 (77.8)
Anishinaabe	1 (7.1)	1 (16.7)	
Denesuline	1 (7.1)		1 (11.1)
Nakota	1 (7.1)		
Other (Anishinaabe/Dakota)	1 (7.1)	1 (16.7)	1 (11.1)
Marital status: *n* (%)			
Single/never married	10 (71.4)	5 (83.3)	5 (55.6)
Married/cohabiting	3 (21.4)		3 (33.3)
Separated/divorced	1 (7.1)	1 (16.7)	1 (11.1)
Year of study: *n* (%)			
First	5 (35.7)	3 (50)	3 (33.3)
Second	2 (14.3)		1 (11.1)
Third	3 (21.4)	2 (33.3)	2 (22.2)
Fourth	3 (21.4)	1 (16.7)	2 (22.2)
Doctoral	1 (7.1)		1 (11.1)
First Language: *n* (%)			
English	13 (92.9)	6 (100)	9 (100)
Other (Cree)	1 (7.1)		
Religious Tradition: *n* (%)			
First Nations/Native Spirituality	9 (64.3)	4 (66.7)	6 (66.7)
Christianity	1 (7.1)	1 (16.7)	
Buddhism	2 (14.3)		2 (22.2)
Other (First Nations/Native Spirituality/Christianity)	1 (7.1)	1 (16.7)	
Other (First Nations/Native Spirituality/agnosticism)	1 (7.1)		1 (11.1)

### Procedure

Phases 1 and 3 of recruitment and item administration were hosted on Qualtrics online surveying system, while the Indigenous Talking Circle was hosted on Zoom videoconferencing technology. Participants in Phases 1 and 3 accessed the survey items by clicking on a link provided via blinded emails. Participant responses from Phases 1 and 3 were linked via an anonymized participation code. All those participating in Phase 1 were invited to attend the Phase 2 Zoom Talking Circle. Those confirming their attendance were provided with the appropriate Zoom link for the Talking Circle. The Talking Circle was led by a Bachelor-level student researcher, and co-led by a First Nations Elder, and a researcher with extensive expertise in Indigenous research methods. The Talking Circle was 1 hr in length and was audio and video recorded (with permission from the Elder and the participants).

Phase 1 was deployed early in February 2021, and participants had 4 weeks to complete the Phase 1 survey. Phase 2 (Zoom Talking Circle) was scheduled for March 4, 2021. Phase 3 commenced on March 19, 2021, and participants had 2 weeks to complete the Phase 3 survey.

### Measures

#### Phase 1: Evaluation of a Generic Mindfulness-Based Intervention Description

In Phase 1, participants were first asked to complete a demographic information questionnaire with questions related to gender, age, Indigenous status, marital status, religious/spiritual belief, academic year of study, program of study, first language, and involvement with mindfulness and/or psychotherapy.

Participants were presented with a brief description of “Depression” and “Anxiety” in accordance with the DSM-5 (American Psychiatric Association, 2013). The description provided participants with information about the nature and typical duration of depression (e.g., “depression can mean a deep sadness or feeling down, or even losing interest in things you usually enjoy”) and anxiety (e.g., “anxiety can mean the experience of fear or panic that you experience in your body and mind”). Participants were then presented with various open-ended questions which asked “What does depression mean to you?”, “What does anxiety mean to you?”, “In your opinion, what may cause depression?”, “In your opinion, what may cause anxiety?”, and “In your view, what are the ways for someone to manage depression/anxiety?”. These questions were adapted from previous studies examining cross-cultural conceptualizations of psychopathology (Lauber et al., 2003; Shellman et al., 2007; Ward et al., 2014; Watson & Beshai, 2021). The questions do not assume a meta-perspective or cultural framework through which to understand depression and anxiety, while eliciting idiosyncratic and lived-experience narratives of such conditions (Ward et al., 2014).

Participants were then invited to read a brief (380-word) description of mindfulness (e.g., “Mindfulness can be defined as purposely or intentionally paying attention to present-moment experiences … with a sense of openness, acceptance, or surrender”) and mindfulness-based programs (e.g., “Mindfulness-Based Programs include weekly sessions, and each program usually spans 8 weeks (depending on the type and format) … sessions are guided by a trained facilitator who guides the participants through several mindfulness meditations and exercises”) (Del Rosario & Beshai, 2022). The description also included a list of evidence-based advantages (e.g., “Mindfulness training has been linked to benefits for anxiety and depression”) and disadvantages (e.g., “Not everyone responds the same to these programs; It is not clear who specifically would benefit most from mindfulness”) of mindfulness-based programs. The description was prepared in keeping with the five essential elements of mindfulness-programs extrapolated by Crane et al. (2017). The content of the description of mindfulness and mindfulness-based programs was vetted by three experts with specialization in mindfulness with 5 + years of experience. These experts read the description and provided feedback regarding its consistency with their understanding of mindfulness and MBIs, and how the description can be improved for clarity and comprehension (Del Rosario & Beshai, 2022).

Participants were then presented with a series of questions regarding how consistent the concept of mindfulness and the generically described mindfulness program are with Indigenous cultures and the culturally sensitive elements and features that should be emphasized in the program. Seven open-ended questions were asked (e.g., “If you have heard of mindfulness, what has been your experience?”, “What does mindfulness mean to you?”, “How would mindfulness be expressed in your culture, worldview or community?”, “What parts of the mindfulness program would you change to make it more consistent with your cultural/spiritual views?”).

Several questions were designed to elicit responses from participants regarding the preferred format and method of delivery of the program and the likelihood of participants incorporating an MBI into their daily lives (e.g., “What would be some of the barriers that would prevent you from starting or attending a mindfulness program?”, “How can a mindfulness program be made more relevant to your lifestyle and schedule as a student?”, “What would be your preferred delivery of a mindfulness program?”, and “What length of time would you recommend for each mindfulness session?”).

#### Phase 2: Talking Circle

In Phase 2, we utilized an Indigenous research method known as storytelling in the form of a Talking Circle. A Talking Circle is a traditional means for communicating information in a manner that is mutually respectful, non-judgemental, and ultimately supportive of each individual in the circle (Kovach, 2009; Rothe et al., 2009). Talking Circles are meant to elicit stories from the participants in a manner they see fit (Kovach, 2009). The aims of the Talking Circle were to (a) provide participants with additional information about mindfulness and mindfulness-programs; (b) obtain a richer understanding regarding the extent of overlap between mindfulness and traditional healing methods, and whether mindfulness is consistent with Indigenous practices and cultures; and (c) gather information on how and which specific elements and features of an MBI could be adapted to make it more culturally relevant to Indigenous peoples and to a university student lifestyle. An Elder was present to clarify any of the content for both the researcher and the student participants. To this end, the lead researcher, who identifies as First Nations (SMD), asked participant attendees various open-ended questions (e.g., “How consistent the concept of mindfulness and the generically described mindfulness program are with Indigenous cultures?; “What aspects of mindfulness resemble aspects of your culture or spirituality?”; “Which culturally sensitive elements and features should be emphasized or incorporated in the program to improve its relevance for yourself ?”; “How important would it be to you that the mindfulness-based program be led by an Indigenous facilitator?”).

#### Phase 3: Evaluation of the Adapted Program Description

In Phase 3, an adapted mindfulness-based program description was developed based on elicited feedback in Phases 1 and 2. In designing this adapted outline, we adopted the Two-Eyed Seeing approach, developed by Albert Marshall (Mi’Kmaq Elder). The Two-Eyed Seeing approach posits that the strengths of the Indigenous and Western ways of knowing and knowledge must both be considered and combined (Iwama et al., 2009). That is, people that such interventions are intended to reach need to be actively engaged in the development and research processes from inception (e.g., generating research questions) through to knowledge dissemination and practical applications (Katapally, 2019). Accordingly, and in collaboration with an Indigenous Knowledge Keeper, we carefully collated, analyzed, and synthesized feedback solicited from students in Phases 1 and 2 with the existing empirical literature related to mindfulness. Student feedback was a primary guide in the decision-making process related to tailoring the hypothetical MBI.

The hypothetical program was titled *Miyowâyâwin Mindful Wellbeing Program*. A description of the adapted mindfulness-based program was presented to the participants. Similar to Phase 1, participants were asked a mixture qualitative, open-ended questions designed to elicit feedback regarding any altered perceptions concerning depression and anxiety (e.g., “Has your participation in this study, to this point, helped you gain insight into depression and anxiety? If yes, what have you discovered?”) and mindfulness (e.g., “As it stands, how much do you know about mindfulness or mindfulness-based interventions?”, “What does mindfulness mean to you now?”).

Participants were asked to provide feedback regarding additional elements or features to be incorporated into the adapted program (e.g., “Are there any other elements of Indigenous spirituality or culture that you think should be further incorporated into the Miyowâyâwin program? … please explain.”).

### Data Analyses

Findings from Phases 1, 2, and 3 were categorized and presented accordingly.

The analysis of data from Phase 2 was carried out over six stages of thematic analysis (Braun & Clarke, 2013). First, the recorded data obtained from the Talking Circle were transcribed verbatim by the researcher. The participants’ data were de-identified by assigning a pseudonym to each participant. Only verbal data were captured for analysis. Second, the researcher reviewed the data thoroughly to ensure there was no missing content and to gain familiarity with the data in preparation for coding. Third, a community leader in Indigenous research methods collaborated on identifying themes within the data. Preliminary coding was conducted by selecting key and recurring words and phrases that tied in with the objectives of the study. Fourth, themes were identified from the clustering of the preliminary codes. Survey responses were exported to a Microsoft Word document to facilitate coding of the data. Fifth, a thematic map was used to explore relationships between the elements expressed by the participants’ needs, opinions, and concepts until no additional themes emerged from the survey data. Sixth, the descriptive terms used to identify the themes were finalized. The results from the online survey and Talking Circle informed the creation of the description of a hypothetical adapted mindfulness-based program.

## Results

### Generic Program Description Feedback and Evaluation

Participants defined depression by its association with lack of motivation, overwhelming thoughts, sadness, apathy, loss of interest in things once enjoyed, hopelessness, helplessness, sleep problems, irritability, and negative thoughts. Furthermore, participants reported depression to be caused by negative thinking, substance abuse, trauma, chemical imbalances, loneliness, abusive relationships, life stressors (e.g., loss of a loved one or unemployment), lack of exercise, and social issues. Anxiety was conceptualized as paranoia, worrying to excess, fear, panic, and physiological manifestations, such as sweating, increased heart rate, tension, and an inability to breathe. Participants reported the causes of anxiety to be social situations (e.g., public speaking or meeting people), self-conscious concerns and overthinking, worrying, trauma, stress, and confrontational situations.

Methods identified by the participants for managing depression and anxiety included self-reflection, self-care (i.e., exercise, healthy diet, sleep), connecting with family and friends, counselling, prayer, smudging, meditation, burning ceremony (i.e., writing unwanted thoughts on paper and burning them), art therapy (e.g., beading, painting, and drawing), and Western medicine (e.g., antidepressants). Two of the participants used traditional Indigenous practices to manage depression and anxiety; seven participants engaged in activities that may be conducive to mindfulness, such as meditation, yoga, walking, running, and prayer; three participants utilized therapy and/or medication; and one participant did not have a method for managing depression and anxiety.

Eleven participants indicated they had heard of mindfulness prior to the study; however, three participants had no prior knowledge. Some participants reported that they had only heard the term while others had become aware of mindfulness in therapy. Mindfulness was described by participants as a process that uses the five senses and focuses on breathing, having an awareness of one’s surroundings and being in the present moment, not allowing thoughts to dwell on past or present, and refocusing thoughts away from negative concerns such as loneliness, fears, doubts, and assumptions about the thoughts and actions of others. Participants identified mindfulness as a mechanism for coping with anxiety and depression, achieving more self-knowledge and mental clarity, reducing social concerns, relief of tension, and quieting thoughts. Participants also identified several aspects of Indigenous culture where mindfulness or a mindful experience might be expressed. These included sweats, smudging, pow wows, drumming, gathering Indian medicine, ceremonial dances (e.g., Sundance), and contact with Elders.

Participants identified several barriers that might prevent them from engaging in an MBI: not having a private space to practice mindfulness; not wanting to divulge personal information regarding their struggles; lack of childcare; demands and obligations to family, work and school; the group setting of the program; feeling vulnerable; and anxiety. Participants also emphasized a need for flexibility in scheduling and intervention format, such as provision of evening sessions, drop-in sessions, recorded sessions, or sessions incorporated into class time. An equal number of participants reported preferring a mindfulness intervention online (*n* = 7) or in-person (*n* = 7), while all participants reported the need for a guided or facilitated intervention rather than self-directed. Participants were willing to accept an online mindfulness program due in part to COVID; however, several thought in-person would be preferable. Participants indicated in-person sessions would allow for more personal contact with others. Furthermore, participants reported that the conduct of traditional Indigenous ceremonies would be better in-person than online. Participants offered some suggestions for making the intervention more relevant to the needs of Indigenous students: a facilitator with knowledge of traditional Indigenous practices, Talking Circles, and prayer and smudging led by an Elder. As expressed by student B, storytelling is another aspect of Indigenous knowledge that could be incorporated into the mindfulness program, “Stories of Nanabush … talk about relationships and feelings that may help a person become more aware of themselves.” Nine participants reported that they would feel comfortable asking other Indigenous students to participate; however, two participants stated they would not feel comfortable asking others, and three participants would not recommend it due to not having contact with friends.

### In-Depth Adaptation Discussion

Themes of access, authenticity, ceremony, and holistic health were identified from an analysis of the Talking Circle transcript. Table 2 provides a summary of the themes and their meanings.

**Table 2:** Identified themes and their meanings

Themes	Meanings	
Access	• Flexible scheduling • Recorded sessions • Contact with Elders for guidance	• Knowledge of traditional plant medicine • Availability of traditional plant medicine
Authenticity	• Terminology reflective of Indigenous culture • Recognition of Indigenous concepts	• Inclusion of Indigenous practices • Employing an Indigenous facilitator
Ceremony	• Sweats • Smudging • Prayer • Fasting • Talking circle • Inclusion of Elders	• Drumming • Pow wow, Sun Dance • Gathering of traditional plant medicine • Recognition of connection to the earth and other people
Holistic health	• Being present in the moment • Mindfulness • Meditation • Counselling • Western medication • Connection with community, culture, and environment	• Connection to the Creator • Self-care • Physical exercise (e.g., running) • Healthy diet • Physical, mental, spiritual, and emotional health in balance

This table shows the themes and their meanings were derived from the input from participants. The themes represent the overarching important requirements for an intervention to be culturally relevant, while the meanings represent the specific elements for inclusion

#### Access

Several students spoke about the problem of accessing health services during the COVID pandemic and the need for mental health supports, such as a mindfulness intervention.

Student B noted that MBIs “need to be promoted and understood more so that a person can find easier access.”

Another frequently mentioned barrier to managing one’s mental health was lack of childcare. Participants, particularly those with family commitments, indicated that they require more flexibility with pre-recorded, online, 20-min sessions. Participants reported that obligations to family, jobs, and university coursework place constraints on time for many; thus, the typical 8-week format of the intervention may not be manageable for many of them.

Another barrier to managing mental health was the lack of access to Indigenous plant medicine for smudging and also learning how to gather and use the plants. Both Student C and Student E indicated that the inclusion of smudge kits and access to Elders should be important considerations in a mindfulness intervention.

#### Authenticity

Authenticity is a key feature of a successful mindfulness intervention that is tailored for a specific cultural group. Authenticity is determined by how closely the mindfulness program adheres to Indigenous cultural values, and by the extent to which it captures the genuine insights and worldview of Indigenous people.

One point raised by participants was that the terminology of the program (i.e., mindfulness) needed to be made more reflective of Indigenous practice by perhaps using terms from some of the languages of the Treaty 4 region. Mindfulness is not a culturally relevant term for Indigenous people and as Student C stated, “the terminology kind of makes it hard to connect with.” Furthermore, participants indicated a need for the program to recognize Indigenous concepts of balance between the physical and social environment and also the four elements (mental, physical, emotional, and spiritual).

Several participants were forthcoming concerning the specific background and understandings of intervention leaders (facilitator and Elder). Inclusion of Elders’ prayers and smudging and use of Talking Circles were identified as important features that would influence a successful mindfulness intervention for Indigenous students. Student C indicated that the program facilitator should be engaged in traditional practices on a deeper level, and they need to operate from the standpoint of a shared experience:

I feel … it might be … genuine, if it was someone that was … rooted in the culture and understandings rather than someone who … read about it. … They have that experience, … shared knowledge and … connection.

For these reasons, participants reported a strong preference for an Indigenous facilitator to guide the intervention. This could work to further integrate Indigenous elements into MBI. Student F expressed the need to keep the program based in Indigenous culture to maintain the connected and balanced approach.

#### Ceremony

Westernized mindfulness is focused primarily on the self, contrary to Indigenous culture where mindfulness finds expression through ceremony, community, and a connection to the earth. There was consensus among participants that mindfulness in the context of the adapted program be expressed through sweats, pipe ceremonies, fasting, smudging, drum ceremonies, pow wows, contact with Elders, and prayer. Moreover, mindful experiences can even be cultivated through gathering Indian medicine wherein the close connection to Mother Nature can be curative. In this framework, mindfulness can be conceptualized as an attitude of appreciation and gratitude towards the Creator/Mother Nature and towards others in the community. It is having respect, kindness, and an open acceptance of other people.

Student E emphasized the importance of including cultural elements into the MBI:

Colonization … took a lot of our culture and our traditions away. … First Nations students … come out into … the urbanized world and then they’re kind of taken away from their culture even that much more. So, I think … a mindfulness program that incorporates … the cultural aspects, keeps you more connected and rooted to yourself.

Participants recommended incorporating traditional Indigenous knowledge into an MBI that would include ceremony in daily life through the inclusion of Elder’s prayers, smudging, and Talking Circles.

Learning about the medicines and how to gather them, while emphasizing the spiritual aspects of gathering and using the medicines, is another way to incorporate traditional knowledge into the intervention. Student E explained how important plant medicine is for mindful practice and how difficult it is to access them without knowledge of the locations of the medicines and the protocols for gathering. Furthermore, Student E identified a need for smudge kits to be made available for their mindfulness practice.

In addition to more knowledge about the medicines, more learning about the history and culture of Indigenous peoples, including art and handicraft as a part of ceremony, was a cultural element suggested by the participants. Recommendations from participants focused primarily on the need to incorporate recognition of connections to the earth and to other people.

#### Holistic Health

An MBI for Indigenous students needs to incorporate a holistic approach to health. Holistic health is an integral component of the Indigenous worldview (Sasakamoose et al., 2017). The mental, emotional, and spiritual elements are each equally as important as the physical (Sasakamoose et al., 2017). It is a complex schema in which relationships with other people and with the Earth come into play.

Several students spoke of a struggle to maintain mental health in the face of isolation from family and community. Ideally, an MBI would incorporate elements of community. As Student C noted, “That [Talking Circles with Elders] would be so beneficial [because] there’s so many people that feel like they’re … alone … [and need to] be able to share how they feel and … their struggles.” Spiritual elements of holistic health, in particular ceremonies, should also be incorporated into the mindfulness-based program. Student C remarked “to be able to have that smudge … when you’re isolated like that, that’s like a saving grace for me … to reach it and to say a prayer.”

Using the feedback obtained from participants during the survey and Talking Circle in Phases 1 and 2, the research team created a program description for a hypothetical adapted mindfulness-based program titled *Miyowâyâwin: Indigenous Mindful Wellbeing Program* (Table 3). This program, which is based on the mindfulness-based stress reduction program, combines mindfulness meditation with Indigenous healing practices and ceremonies to make it more suited to the culture and lifestyle of an Indigenous student.

**Table 3:** *Miyowâyâwin*: Indigenous Mindful Wellbeing Program description

Intervention description	Mindfulness is paying attention to present moment experiences with purpose, openness, and non-judgment. The *Miyowâyâwin: Indigenous Mindful Wellbeing Program* is specifically designed for Indigenous-identified university students struggling with symptoms of depression and anxiety. Using a blend of mindfulness meditation and Indigenous healing practices and ceremonies, this program aims to help students improve their mental wellbeing by changing their relationship to the present moment. In the context of this program, students will learn to pay attention to things such as the breath, bodily sensations, sounds, smells, and sights. Guided by **a trained Indigenous mindfulness facilitator**, students will learn to pay attention to unfolding experiences in the context of traditional ceremonies, such as **smudging (e.g., paying close and open attention to smells and other sensations arising during the experience), sweat lodges (paying close and open attention to the sensations and sights of the open flame), and traditional drumming (paying close and open attention to sounds and rhythms).** This will be combined with mindful meditation practice where students will learn to pay close attention, in an accepting way, to sensations of breathing, the body, and contents of mind (e.g., thoughts, emotions). The intervention will emphasize **holistic integration of the spiritual, mental, emotional, and physical** aspects of existence
Structure of intervention	The intervention will be delivered **in-person** (assuming it is safe to do so), within a **group format (8–10 students per group),** and will be **5 weeks in duration**. Each session will be 45 min in duration, and each will open with a **traditional prayer**. Each group will be invited to take part in two talking circles, led by an Elder and the trained facilitator. The first will commence prior to starting the first week of the intervention, while the second will take place shortly after the last week of the intervention. The talking circles will act as a way to openly discuss concerns and expectations for the intervention (Talking circle 1), as well as consolidate learning and debriefing (Talking circle 2)
Delivery model	The intervention is designed to be flexible. Each weekly session will be recorded (with permission from the facilitator and students), and **recordings of each of the sessions will be accessible online** for those who miss one of the weekly sessions, or those who want additional access to the group sessions or teaching contained within. Participants will be invited to complete between-session exercises and meditations designed to further develop skills learned during the weekly in-group sessions
Facilitator	The **facilitator will be Indigenous**. They will be familiar with traditional mindfulness approaches (e.g., Mindfulness-Based Stress Reduction), as well as familiar with Indigenous healing practices and cultures. The exercises and teachings contained within the intervention will be closely monitored and approved by an Elder

### Adapted Program Description Feedback and Evaluation

Findings reveal that 8 of the 9 participants completing Phase 3 indicated they had gained new insight into depression and anxiety as a result of their participation in the study. Some participants discovered that sharing with others helped them cope and showed them that they were not alone in their struggles, that others dealt with similar issues. Student B stated that participation in the study had brought about an awareness of their own experience with depression and anxiety and how they might manage the symptoms of depression and anxiety. Student M remarked on the value of mindfulness and traditional Indigenous practices in alleviating the symptoms of depression and anxiety:

I did not think about how colonization would affect Indigenous peoples’ mental health and how we could combat this using mindfulness (which also has been influenced by colonization) while still incorporating traditional aspects. I assumed that to help with my depression and anxiety, I would have to adapt to a Western medical practice, however this program would allow Indigenous peoples to exercise traditional practices.

Several other participants underscored the words of Student M that traditional ceremony and mindfulness could help alleviate their anxiety.

Eight of the nine participants reported an increased understanding of mindfulness since the beginning of the study; one participant reported no increase. Participants reported their new understanding of mindfulness as “an opportunity to slow down” and “be present in the moment.” Student E described her recent experience with mindfulness: “as I was stressed about the end of the term, I was able to remind myself of mindfulness and enjoy the class lecture, rather than think about the time left.” Student M, who had been treated with therapy and medication, came to the realization that “mindfulness can extend beyond the Western ideas it is based in, and can help the healing process by incorporating Indigenous aspects.”

Some of the benefits of the Miyowâyâwin program, as identified by the participants, included the presence of an Elder; having a trained Indigenous facilitator; an opportunity to participate in traditional Indigenous ceremonies; connection with others who have a shared history and experiences; and acquiring strategies for coping with depression and anxiety. For example, Student H noted the benefits of the Miyowâyâwin program as:

Learning good ways to help deal with stress, anxiety, and depression. Utilizing our Indigenous ways of knowing and being, along with what we learn in the program.

Participating with like-minded people, who understand and know what we face.

Participants identified additional features of Indigenous spirituality or culture that could be incorporated into the intervention: role of prayer, dance or movement, storytelling, arts and crafts (e.g., dreamcatchers, medicine bags), access to plants (i.e., sage, sweetgrass) for home practice, and space (e.g., a quiet area with low lighting such as a tipi).

Participants (*n* = 9) reported that they would feel comfortable sharing information about the Miyowâyâwin program with other Indigenous students.

Student B identified a need for support for those who might have “struggles to maintain mindfulness at home” beyond the 5-week duration of the Miyowâyâwin program.

## Discussion

In the current investigation, we examined perceptions of Indigenous students enrolled in a medium-sized university in Canada on the consistency of mindfulness and mindfulness-based interventions with Indigenous cultures and perspectives. We also solicited feedback from these students on how to adapt a traditional mindfulness-based intervention to be optimally consistent with Indigenous worldviews and student lifestyles. A mixture of qualitative and Indigenous methodologies (Talking Circle) revealed high acceptability and credibility perceptions of mindfulness and mindfulness interventions among Indigenous students. Results also indicated Indigenous students would value an adapted mindfulness intervention that (a) is flexible and accessible; (b) blends mindfulness teachings with Indigenous concepts, narratives, and worldviews; and (c) cultivates the capacity for mindfulness through Indigenous ceremony (e.g., Sweats; Smudging; Prayer; Indigenous art and storytelling).

Indigenous university students experience higher rates of depression and anxiety (Chahar Mahali et al., 2020; Kirmayer et al., 2000) due to the compounding effects of academic pressures, family obligations, racism, discrimination, and the ongoing effects of colonialism. Unfortunately, there is a lack of culturally relevant mental health services for Indigenous people in general (Gone, 2004; Hodge et al., 2009) and there have been some circumstances of abuse and neglect that have resulted in a lack of trust on the part of Indigenous peoples (Tang & Browne, 2008). MBIs can help alleviate anxiety and depression but existing programs are not grounded on Indigenous cultures, despite mindfulness being an element of Indigenous spiritual practice (Yellow Bird, 2012).

Access to, availability, and cultural relevance of mental health services are social justice issues. To ensure health equity and to tackle the ever-growing problem of health disparities in our society, appropriate services should be made available to all peoples. There is a paucity of literature relating to MBIs for Indigenous people, and more particularly for Indigenous university students. Most of the studies have been carried out in Western, Judeo-Christian contexts. This study sought to address this gap in the literature. Using a mixture of methodologies, we sought to gather feedback regarding acceptability and cultural consistency of mindfulness and MBIs among Indigenous students. Importantly, in this study, we gathered feedback regarding the specific cultural and lifestyle needs of Indigenous students, and how to best adapt an MBI for depression and anxiety to meet such unique needs.

We found that Indigenous participant narratives of depression and anxiety were mostly consistent with the DSM definition of the constructs (e.g., depression: sadness, apathy, presence of negative cognitions; anxiety: fear, panic, tension; American Psychiatric Association, 2013). They reported a diversity of biopsychosocial factors that were perceived to account for the development of depression and anxiety. While their definitions of depression and anxiety overlapped greatly with DSM definitions, they emphasized traditional healing methods in addition to conventional Western methods as ways of coping with these conditions. This is consistent with previous literature highlighting cross-cultural differences in coping strategies, wherein certain cultures emphasize the incorporation of spirituality and connectedness in dealing with mental health concerns (Beshai et al., 2013).

While participants rated their knowledge of mindfulness as low to moderate, their concept of mindfulness emphasized present-moment awareness of one’s environment. Accordingly, even these relatively mindfulness-naïve participants could identify the centrality of the present-focused attentional dimension of mindfulness (Bishop et al., 2004). When participants were presented with the unadapted program description, students were still able to identify a correspondence with Indigenous culture. Mindfulness was found to be a component of traditional Indigenous practices, suggesting mindfulness shares common elements with traditional Indigenous practices and principles (Sasakamoose et al., 2016; Yellow Bird, 2012).

Participants were unanimous in their desire for a flexible MBI that takes into account competing demands of family, work, and university. In particular, a shortened program was preferred over the traditional first generation MBI format of 2 hr per week with an 8-week duration and substantial time commitment to between-week practice (Crane et al., 2017). Second-generation interventions have employed this level of flexibility without necessarily sacrificing efficacy (Van Gordon et al., 2015).

While some participants emphasized the need for in-person Indigenous ceremonies as part of the adapted intervention, others also saw value in an online format, given its accessibility. These findings are consistent with findings from previous research which recommends making accommodations to suit the needs of the target population and thus increasing engagement with a program. Some of these methods include shortened sessions (Woods-Giscombé & Gaylord, 2014) and holding sessions at a convenient time of day (Le & Gobert, 2015).

Most participants expressed approval for the unadapted intervention, such that they would attend or would recommend it to others. The majority of participants found the unadapted MBI description consistent with Indigenous culture. Furthermore, most participants indicated that they would likely participate in or attend the unadapted MBI for depression. Although participants overall expressed support for the unadapted MBI, they also indicated adaptations were required to make it more consistent with their Indigenous culture and student lifestyle (Proulx et al., 2020; Watson-Singleton et al., 2019; Woods-Giscombé & Gaylord, 2014).

Indigenous participants emphasized the importance of the cultural authenticity of the adapted MBI that captures genuine insights, worldviews, and ways of knowing of Indigenous people. For example, participants suggested that the adapted MBI should include terminology and content that would reflect Indigenous cultures. Previous findings also highlight the need for cultural matching of facilitator to cultural target of intervention adaptation, and the incorporation of culturally relevant terminology in the content of the intervention (Salamanca-Sanabria et al., 2019; Watson-Singleton et al., 2019).

The importance of ceremony was emphasized by participants in all phases of data collection. Ceremony is woven like a strong thread throughout traditional Indigenous community life, and there was a consistent recognition by participants of the need for such ceremony. The need for traditional Indigenous practices—smudging, prayer, and guidance from Elders—was emphasized as critical components for the adapted MBI, and as direct means to cultivate mindfulness. Cross-cultural intervention adaptation literature reliably emphasizes the importance of incorporating spiritual beliefs of the community in adapted manuals (Bernal et al., 2009; Proulx et al., 2020; Watson-Singleton et al., 2019).

The participants’ understanding of holistic health was represented by the balance reminiscent of a medicine wheel with its four elements: care for the physical body through nutrition, rest and exercise; social contact, especially with home and community; balancing emotion and thought through self-awareness and connection to nature; and making a spiritual commitment with prayer, smudging, sweats, drumming, and other features of spiritual practice. The spiritual component was foremost, with particular emphasis on the need for ceremony (Beshai et al., 2012, 2013). This finding is consistent with literature suggesting the importance of incorporating cultural values in mental health interventions (Green, 2010). This adaptation functions to improve the cultural relevance of the interventions, and hence has potential to improve engagement and outcomes.

In Phase 3, participants expressed gaining new insights into the value of mindfulness in managing depression and anxiety, and they had raised their awareness of the extent to which colonialism played a role in mental illness among Indigenous peoples. Despite its connection with Western approaches, some participants found that mindfulness could be used to combat the pernicious effects of colonialism. The outline for the adapted MBI was identified as particularly beneficial in providing contact with others who have a shared experience of colonialism and its impacts. Student participants reported that the researchers were receptive to their feedback in designing the outline for the adapted MBI, and that the researchers were successful in incorporating the elements they originally identified as critical in the MBI adaptation process for optimal cultural relevance.

Consistent with hypotheses, Indigenous students found the adapted mindfulness-based program description to have a high degree of consistency with Indigenous cultures, and reported the program as acceptable. This finding is consistent with previous findings suggesting efforts to culturally adapt generic treatments for mental health conditions improve patients’ perceptions of the social relevance and credibility of such treatments (Huey et al., 2014). While participants perceived significant consistency of even the unadapted version of the mindfulness intervention description with Indigenous worldviews, the results of the current investigation still highlight the need for further tailoring to optimize cultural relevance and safety of the interventions. For example, developers of first generation mindfulness-based interventions were deliberate in their exclusion of the spiritual elements of mindfulness (Crane et al., 2017; Van Gordon & Shonin, 2020). These exclusions reduce the cross-cultural relevance of MBIs among people of cultures that center spirituality, connectedness, and holistic integration (Fleming et al., 2022). Accordingly, and despite the superficial consistency, more deep-seated tailoring is often required to re-integrate these lost elements of mindfulness back into the intervention.

To our knowledge, this is the first study that organically solicits feedback regarding MBIs among Indigenous people in Canada. In doing so, we used a mixed-method longitudinal design, employing Indigenous methodologies with the guidance of a cultural leader to solicit such feedback. There is currently a paucity of research examining perceptions of MBIs among minoritized people. This study helped bridge this research gap by examining acceptability and cultural consistency of MBIs among Indigenous university students, and solicited feedback from said students on how to adapt a generic MBI to make it more consistent with Indigenous cultures, values, and practices.

### Limitations and Future Research

The study had several limitations that pave the way for future studies. First, while the sample might be representative of some Indigenous views, Indigenous cultures are heterogenous, and so could not be represented in their fullness in this adaptation process. Second, the small sample size has limited reliability when drawing inferences about the needs of the larger Indigenous student population in Canada. Third, Talking Circles are typically conducted in-person; however, due to the COVID-19 pandemic, we employed an online format, which lacked the intimacy needed for this particular methodology. Finally, and perhaps due to the mode of the Talking Circle, not all the participants who were present in the circle contributed equally, and so this may have biased responses.

The current study has potential to inform future research by providing a body of knowledge on elements of evidence-based programs that are primed for cultural adaptation. Furthermore, results of this study will be used as foundation to further develop the *Miyowâyâwin Mindful Wellbeing Program*, and then to examine the efficacy of the intervention to reduce symptoms of depression and anxiety among a group of Indigenous students. Studies such as the current are crucial in helping close the health disparity between Indigenous and non-Indigenous Canadians, in keeping with a call to action by the Truth and Reconciliation Commission of Canada (TRC, 2015). Results highlight the need to incorporate cultural elements and facilitators of the target culture in adapted MBIs to improve their relevance and uptake. Procedures employed in the current study could serve as a prototype for mental health researchers interested in working with Indigenous communities, and the opportunity would be there for local community leaders and practitioners to build on the work done in the context of the current study to meet the various needs of their communities.

In this study, we found overlap between mindfulness and traditional Indigenous healing methods. Furthermore, we found participants perceived mindfulness as consistent with Indigenous practices and cultures. Participant feedback was utilized in the adaptation of a generic intervention description to make it more consistent with Indigenous cultures. Acceptability and cultural sensitivity of the adapted MBI description were established. These findings are important since they lend credence to the idea that MBIs can be adapted for culturally diverse groups. These findings also provide a basis for future research in the adaptation and implementation of psychological programs for Indigenous peoples.

## Data Availability

All data are available at the Open Science Framework.
